# *Kazachstania slooffiae*, an emerging pathogen to watch for in humans?

**DOI:** 10.1016/j.mmcr.2023.08.007

**Published:** 2023-08-28

**Authors:** Ana Cristina Gallotti, Mar Lombera, Karen Pinto, Ignacio Pinilla, Oscar Zaragoza, María Soledad Cuétara

**Affiliations:** a Department of Microbiology and Parasitology, Severo Ochoa University Hospital, Madrid, Spain; b Digestive Unit, Severo Ochoa University Hospital, Madrid, Spain; c Department of Anathomopathology, Severo Ochoa University Hospital, Madrid, Spain; d Mycology Reference Laboratory, National Center for Microbiology, Carlos III Health Institute, Madrid, Spain; e Center for Biomedical Research in Network in Infectious Diseases (CIBERINFEC-CB21/13/00105), Carlos III Health Institute, Madrid, Spain

**Keywords:** *Kazachstania slooffiae*, Antifungal susceptibility, Human pathogen, Esophagitis

## Abstract

In an 80-year-old man with long-term dysphagia, an upper endoscopy was performed and biopsy samples collected for microbiological and pathological tests, showing fungal structures. *Kazachstania slooffiae* was isolated in microbiological cultures that were later confirmed with DNA sequencing. Susceptibility tests were performed, and antifungal treatment was initiated with a clinical, pathological, and microbiological response.

## 1 Introduction

The genus *Kazachstania* includes more than 50 ascomycetous yeast species that are ubiquitous. They play an important role in food industry such as wineprocess [1] or in traditional French wheat [2]. *Kazachstania slooffiae* (synonyms: *Candida slooffiae* (1957); *Torulopsis pintolopesii* var. *slooffiae* (1975); *Candida pintolopesii* var. *slooffiae* (1984)) belongs to *Kazachstania telluris* species complex, with other species such as *Kazachstania bovina, Kazachstania pintolopesii, Kazachstania heterogenica* and *Kazachstania telluris* [3]. Most strains from *K*. *telluris* complex have been isolated in the USA and Europe but a few strains from South Africa, South America and Asia [4]. Their distribution is restricted to the nasal passages stomach and intestinal tracts of birds and mammals. The species may have some host specificity [4].

*Kazachstania slooffiae* has been isolated from the horse and porcine gut. The high incidence (48.4%) suggested that the pig rather than the horse might be its natural host [5] and it is therefore considered a natural commensal of the porcine intestinal environment [5,6].

However, fatal cases in animals by *Kazachstania telluris* species complex have been described [7] but little is known about its role as a pathogen in humans.

We report the case of an elderly man who was followed up by the digestive unit for a progressive dysphagia and was initially evaluated with a barium gastroduodenal study that showed a significantly dilated esophagus. An upper endoscopy was requested to obtain samples for microbiology and pathological anatomy. Both studies confirmed an infected esophagitis caused by *K. slooffiae*, a fungus that has been rarely described as a human pathogen to date.

## 2 Case report

**Fig. 1:**
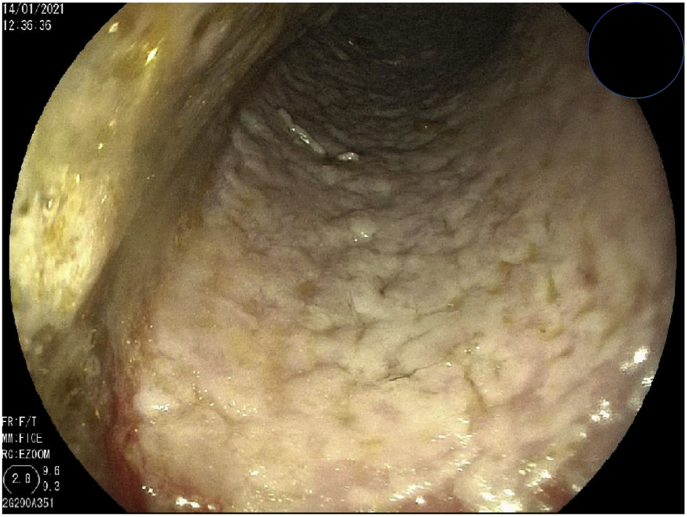
First esophageal upper endoscopy, whitish layer due to the confluence of plaques for fungal infection.

An 80-year-old male, with a previous history of iron-deficiency anemia and prostate cancer treated with hormonal therapy and progressive liquids dysphagia for years, at day 0 went to medical consultation due to worsening of his dysphagia in the last two months, accompanied by loss of appetite and weight loss. He was initially evaluated with a barium gastroduodenal study that showed a significantly dilated esophagus. At day +1 upper endoscopy was performed after 12 h fasting; the esophageal mucosa was covered with a whitish irregular layer, apparently thick (Fig. 1), and stiff to the touch of the biopsy forceps. Aperistalsis esophagus was clearly perceived. A relatively stenotic cardia was seen, but we could surpass it easily, no other distally significant findings.

**Fig. 2:**
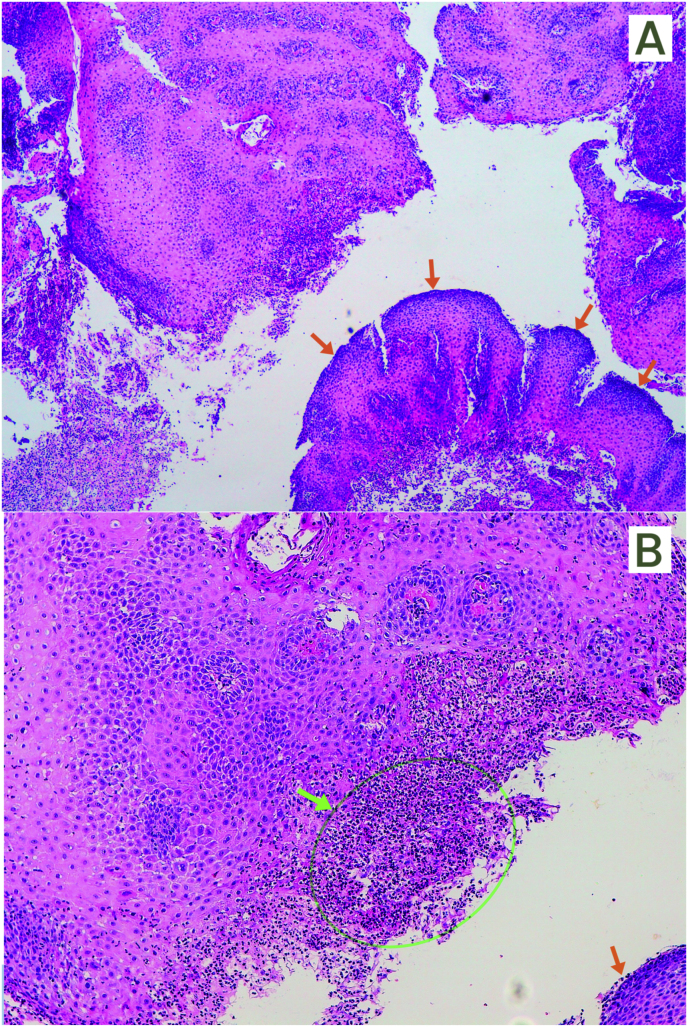
HE staining of biopsies from esophageal mucosa with reactive hyperplasia (orange arrow) and polymorphonuclear infiltrate (green arrow). A) 4x. B) 10x

**Fig. 3:**
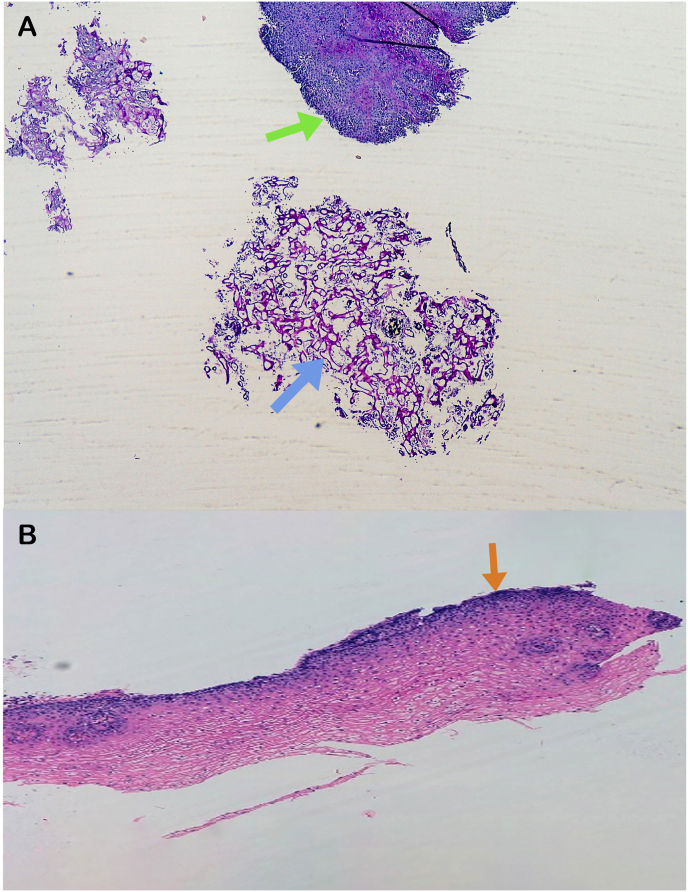
A) PAS staining of biopsies from esophageal mucosa before treatment (4X). Green arrow denotes polymorphonuclear infiltrate; blue arrow denotes fungal structures. B) HE staining of esophageal mucosa biopsies after one month of treatment with fluconazole (10X). Orange arrow denotes hyperplasia. (For interpretation of the references to color in this figure legend, the reader is referred to the Web version of this article.)

Biopsies of the esophageal mucosa demonstrated a paved non-keratinizing mucosa with a polymorphonuclear inflammatory infiltrate (Fig. 2A and B), and the PAS stain showed fungal structures (Fig. 3A).

**Fig. 4:**
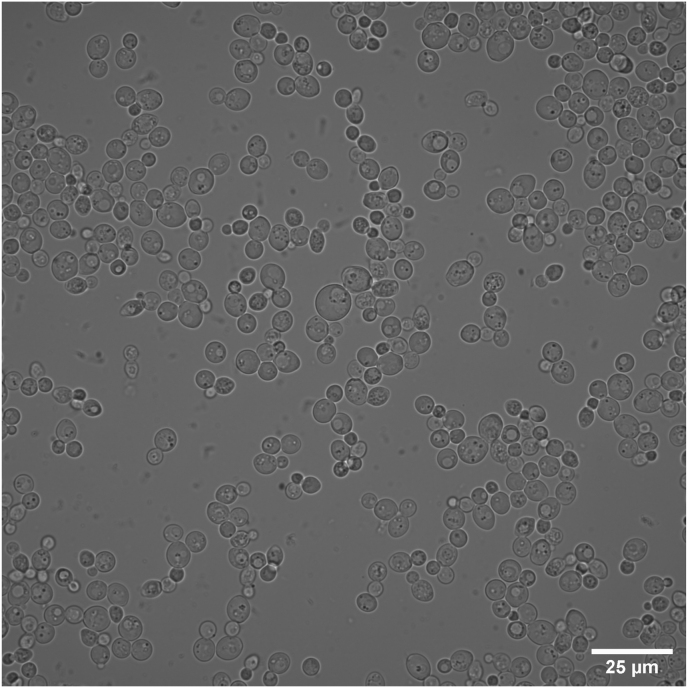
Direct microscopy shows spherical yeast cells with budding.

The biopsy sample was cultivated on Sabouraud dextrose agar plate and incubated at 35 °C. Colonies were shiny and cream-colored with lobate margins. On the microscopy, yeast cells were spherical to ellipsoidal and had multilateral budding and lacking true hyphae (Fig. 4, supplemental video 1). Identification was performed with the MALDI-TOF MS analysis of the MALDI Biotyper instrument equipped with MALDI Biotyper software version 4.1.14, obtaining *Kazachstania slooffiae* score of 1.71.

Supplementary video related to this article can be found at https://doi.org/10.1016/j.mmcr.2023.08.007

The following is/are the supplementary data related to this article:

Multimedia component 1 Supplemental video 1. Cell division of *K. slooffiae* on Sabouraud (left) and RPMI supplemented with 2% glucose and buffered at pH 7 with MOPS at 30 °C (right)1 Multimedia component 1

**Fig. 5:**
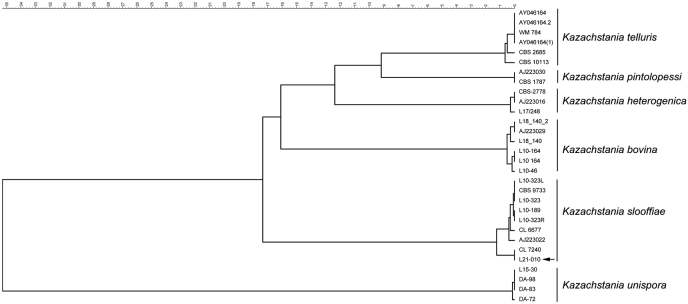
Phylogenetic tree based on the ITS sequence of *Kazachstania* spp.

Fungal identification was confirmed in the Mycology Reference Laboratory of the National Centre for Microbiology by sequencing the internal transcribed sequence from the ribosomal DNA [8]. ITS region was amplified by PCR using ITS1 (5′TCCGTAGGTGAACCTGCGG3′) and ITS4 (5′TCCTCCGCTTATTGATATGC3′) oligonucleotides. Finally, the PCR products were sequenced using ITS1 and ITS4 oligonucleotides using the Sanger protocol. Correct identification was confirmed by comparison of the sequence in the Nucleotide Blast Data Base (NCBI) and by using the database of the ITS sequences of the Mycology Reference Laboratory of the National Centre for Microbiology using Bionumerics software (version 8.1.1, BioRad). A phylogenetic tree using UPGMA with Kimura correction for multiple alignment was generated. As shown in Fig. 5, ITS sequence discriminated between the different *Kazachstania* spp, confirming the identification of *K. slooffiae* for this isolate. The ITS sequence from the isolate described in this article (L21-010) has been deposited in GenBank with the accession number OR258298.

Even though, no specific breakpoints have been established for the antifungal susceptibility of *Kazachstania* spp., antifungal susceptibility testing was performed using Sensititre® YTAMYUCC panel (Thermofisher® Scientific). The pathogen showed good antifungal susceptibility with the following MICs (μg/mL): amphotericin B 0.25, fluconazole 0.25, posaconazole 0.015, voriconazole 0.008, itraconazole 0.015, isavuconazole 0.008, anidulafungin 0.06, micafungin 0.03 and caspofungin 0.125. Susceptibility could not be performed using EUCAST protocol since this isolate did not grow in RPMI medium **(supplemental video 1)**.

The diagnosis of infectious esophagitis due to *K. slooffiae* was made and the patient underwent an oral fluconazole treatment for 21 days (400 mg first day and 200 mg/d for the remaining 20 days), after which he described an important clinical improvement and no longer complained of dysphagia.

An esophageal manometry was done, and surprisingly it was not compatible with a proper achalasia-because the IRP (integral relaxation pressure of the LES – lower esohageal sphincter-) was not high, it was below normal but a complete esophageal aperistalsis was registered in every swallow. In spite of these findings, the patient assured he had clinically improved in his usual condition.

**Fig. 6:**
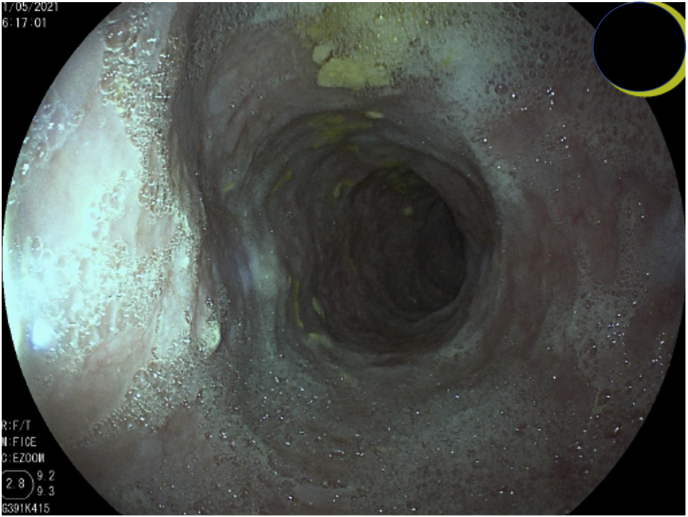
Esophageal image in the control upper endoscopy, one month after finishing fluconazole treatment.

An upper endoscopy was repeated a month after the end of the treatment (Fig. 6) and although the esophageal dilatation was still present, biopsies both for histology and microbiology studies were taken. No more fungal structures were seen in the anatomopathological examination (Fig. 3B) and microbiological cultures were negative.

## 3 Discussion

Invasive fungal diseases present a changing epidemiology, in which more fungi are becoming a threat for human health, which is mainly due to the increase number of immunosuppressed patients which are at risk of suffering opportunistic infections. In this case, we describe a case of esophagitis caused by the yeast, *K. slooffieae*, which is an uncommon human pathogen.

To our knowledge, the first human case published was a 50-year-old shepherd hospitalized in the intensive care unit for a hiatal hernia complicated by an occlusive syndrome in post-surgery and with acute respiratory distress due to mediastinitis with large pleural effusion. *K. slooffiae* was isolated in the pleural sample, and sensibility testing could not be performed. The patient was treated with caspofungin for six weeks showing good recovery [9].

In the same year another case was described in France [10], although MALDI-TOF and the Vitek MS 3.2 system (bio-Mérieux) identified it as *K. slooffiae*, DNA sequencing confirmed *K. bovine*. The case is interesting because it corresponded to a 94 year old male with no history of interest, during his hospitalization for a fall, he presented digestive disorders with postprandial regurgitations and malnutrition. A dolicho-mega esophagus and suspected achalasia were confirmed and he underwent endoscopic pneumatic dilation to treat achalasia. The following day, as a result of fever, blood cultures were taken and *Streptococcus mitis/oralis* and a yeast were detected. Although at first thought to be a *K. slooffiae*, was eventually confirmed as *K. bovine*, and he received caspofungin until 14 days after the negative blood culture. Both species belong to *K. telluris* complex.

A retrospective observational study in Strasburg [11], France was released in 2021 where 13 patients had *Kazachstania* spp. positive samples from 2007 to 2020, of which 9 were considered colonization (no antifungal treatment was administered), and 4 were considered proven fungal infections (1 case of fungemia and pyelonephritis, 1 mediastinitis, 1 esophagitis and 1 angiocholitis). In these four patients with proven infection, species different from *K. slooffie* were isolated. The first three cases were mixed infection by *K. bovine* and *C. albicans*; the first case was treated with fluconazole + caspofungin, the second with caspofungin and the third with esophagitis on esophageal achalasia with only proton pump inhibitor and peroral endoscopic myotomy to treat achalasia without any antifungal. The last case, a recurrent angiocholitis by *K. telluris* complex on the cephalic duodenopancreatectomy was treated with fluconazole and gastrogastrectomy and hepatico-jejunal and gastrointestinal anastomosis. All of proven infection were in conjunction with *C. albicans*, the reason for which it is not possible to determine the contribution of each pathogen to the disease [11].

In our case, *K. slooffie* was the only microorganism isolated and observed in the esophageal biopsy. For this reason, the patient was started with fluconazole. Antifungal susceptibility testing of *K. slooffiae* is challenging, due to the poor growth of these isolates in RPMI medium, so more studies are warranted to evaluate the susceptibility profile of this species.

Based on the optimal clinical-histological-mycological response we believe that this yeast was responsible for the clinical presentation of our patient. More importantly, the patient has been periodically followed up and to date, he has not presented any recurrence of esophagitis.

The incorporation of MALDI-TOF MS into the clinical laboratory has undoubtedly been of great help, however, as far as the identification of fungal pathogens is concerned, we agree with other authors that the discrimination between species of the *Kazachstania telluris* complex remains problematic [11,12] and should be confirmed with complementary techniques, such as the sequencing of the ITS region of the ribosomal DNA.

At this point, it is important to highlight that *K. slooffiae* belongs to the ancient *Candida slooffiae* species and although we have not found *C. slooffiae* esophagitis in the literature, it is also true that on many occasions the diagnosis of candidal esophagitis is made on the basis of the clinical presentation and endoscopic images without identifying the pathogen responsible, so we may be facing an under-diagnosis.

It is noteworthy that in the cases described by *K. slooffiae* or *K. bovina* they presented digestive disorders or hiatal hernia with post-surgical occlusive symptoms [9] or postprandial regurgitations due to dolichomega esophagus and achalasia in the latter [10] or esophagitis on esophageal achalasia [11], similar to symptoms of the patient described in the present article.

The potential role of this new species as human pathogen rises several questions such as: differentiation between proven infection and colonization and the importance of correct identification by DNA sequencing and antifungal susceptibility (this is a challenge in many laboratories due to the scarcity of resources for mycology in many laboratories). For this reason, reporting of future cases will be of importance to confirm *K. slooffiae* as a human pathogen.

## Ethical form

Written informed consent was obtained from the patient or legal guardian(s) for publication of this case report and accompanying images. A copy of the written consent is available for review by the Editor-in-Chief of this journal on request.

## Funding

O.Z. is funded by grant PID2020-114546RB by MCIN/AEI/10.13039/501100011033 (Spanish Ministry for Science and Innovation).

## Ethical statement

Written informed consent from the patient has been obtained from the patient.

## Declaration of competing interest

Authors declare no conflict of interest.
